# Editorial: AIDS 40th Year

**DOI:** 10.3389/fmicb.2023.1184684

**Published:** 2023-03-31

**Authors:** Jiasheng Zhou, Shibo Jiang, Tongqing Zhou, Zhiwei Chen, Xia Jin, Wenyan Zhang, Supachai Rerks-ngarm, Anna Kramvis, Kai Deng, Linqi Zhang

**Affiliations:** ^1^Key Laboratory of Tropical Diseases Control, Zhongshan School of Medicine, Sun Yat-sen University, Guangzhou, China; ^2^Key Laboratory of Medical Molecular Virology (MOE/NHC/CAMS), School of Basic Medical Sciences, Institute of Infectious Disease and Biosecurity, Fudan University, Shanghai, China; ^3^Vaccine Research Center, National Institute of Allergy and Infectious Diseases, National Institutes of Health (NIH), Bethesda, MD, United States; ^4^School of Clinical Medicine, Li Ka Shing Faculty of Medicine, AIDS Institute, The University of Hong Kong, Hong Kong, Hong Kong SAR, China; ^5^Shanghai Public Health Clinical Center, Fudan University, Shanghai, China; ^6^The First Hospital of Jilin University, Institute of Virology and AIDS Research, Changchun, China; ^7^Department of Disease Control, Ministry of Public Health, Bangkok, Thailand; ^8^Hepatitis Virus Diversity Research Unit, Department of Internal Medicine, School of Clinical Medicine, Faculty of Health Sciences, University of the Witwatersrand, Johannesburg, South Africa; ^9^Comprehensive AIDS Research Center, Global Health and Infectious Diseases, School of Medicine, Tsinghua University, Beijing, China

**Keywords:** HIV-1, AIDS, virology, epidemiology, fungus

On June 1981, case reports from five AIDS patients in California, USA were published in *Morbidity and Mortality Weekly Report*, beginning the 40-year battle between humans and AIDS. In 1996, Dr. David Ho initiated highly effective antiretroviral therapy (HAART), which gradually prevented the rapid spread of HIV in humans and dramatically reduced the number of deaths from AIDS, globally. However, according to the report from the Joint United Nations Program on HIV/AIDS (UNAIDS) published in 2022, “Global AIDS Prevention Progress Report 2022: In Danger,” up to 650,000 people still died from AIDS-associated diseases and approximately 1.5 million people were newly diagnosed as HIV-1 positive in 2021, even though the use of HAART had been established 25 years ago. While the indefatigable efforts from global scientists over the past 40 years have significantly reduced HIV-1 prevalence, a permanent cure for HIV-1 patients is still a long way off and a more comprehensive understanding of HIV-1 is urgently required. Here, 40 years since the discovery of HIV/AIDS, we discuss this topic as a special issue of Frontiers in Microbiology, to provide the scientific community with current understanding of HIV-1.

In total, 18 articles have been published in association with this topic. In this Editorial, we focus on four major areas as follows. First, from the perspective of epidemiology and virology, we explain the principles of viral transmission from different subtypes of viruses as well as their differences in virology and immunological response of the host; second, we highlight what is currently known about the host-HIV-1 interaction in terms of changes in host cell numbers and physiology during HIV-1 infections; third, we summarize the new findings on relationships between HIV-1 infections and other diseases; lastly, we briefly review the newly developed methods for detecting HIV-1/AIDS. Taken together, we hope that this collection of scientific articles provides new outlooks on studying HIV-1 and AIDS from different perspectives as well as providing comprehensive understanding of the specific phases of infection to identify and confirm viral mutations associated with drug resistance. In addition, the positive and negative responses of various host cells toward infection and new strategies to reduce physiological discomfort resulting from primary and secondary infections are also discussed. Ultimately, it is our hope that these new research findings provide new avenues and guidelines to prevent the spread of the virus and stimulate the search for a cure for AIDS, eventually leading to the elimination of HIV-1.

It is critical to understand the epidemiology of AIDS and the spread of HIV-1. From 2010 to 2016, Li M. et al. conducted a seven-year horizontal epidemiological survey in Heilongjiang Province, China. By focusing on CRF01_AE, a main subtype of HIV-1, the study showed that sexual contact between men who have sex with men (MSM) was the major route of HIV-1 transmission. In addition, based on a study of the HIV-1 longitudinal genetic network in Guangxi, China, Chen Z. et al. highlight the role of antiretroviral therapy in preventing the spread of HIV-1. In the meanwhile, from population level studies, the authors noted that elderly male farmers, who were not educated in junior high schools, are at high risk of HIV infections. In a different study, Sivay et al. showed that the virus CRF63_02A6, the prevalent genetic variant of HIV-infected individuals in a major part of Siberia, probably originated from Novosibirsk, Russia, in 2005.

Although the virological aspect of HIV-1 has been studied for a relatively long time, given the evolution of virus-generated mutations, the characteristics of those new virus subtypes need to be clarified as an important reference for subsequent practices including disease prevention and control. In that regard, Li Y. et al. expressed HIV-1 CRF07 BC Gag antigen (p6Δ7 mutated or wild type) and screened the immunogenicity of the respective antigens in mice. They found that the single deleted amino acid within the P6 region of the Gag protein most likely resulted in enhanced immunogenicity of the mutated antigen. Meanwhile, by comparing the sequences of HIV-1 subtype B viruses from China and United States, Qian et al. verified two specific sites in the viruses from China, which contribute to the significant increase in viral mRNA transcription and Rev response element activity.

In the pathology of HIV infections, host related changes profoundly affect the development of AIDS. From the perspective of the relationship between host and HIV-1 virus, studies have reported that multiple cells types play diverse roles in the course of a HIV infection. Immune activation is the key factor leading to the impairment of immune reconstitution after long-term HAART. Double negative T cells (DN T) confer immunomodulation during HIV infections, but the relevant mechanism is still puzzling. In two articles within this special topic, different researchers studied the role of two different DN T cells in immune activation during HIV-1 infection. On one hand, Zhang et al. reported that Foxp3^+^ CD4^−^ CD8^−^ T cells accumulated in untreated people living with HIV (PLWH), and the proportion of those cells was negatively correlated with CD4^+^ T cell numbers and CD4^+^/CD8^+^ ratios, but positively correlated with immune activation and systemic inflammation in PLWH. On the other hand, Wang et al. found that, when compared with Immune Responders (IRs), Poor Immune Responders (PIRs) showed reduced percentages of CD73^+^ CD4^−^ CD8^−^ T cells. The cell frequency was positively correlated with CD4^+^ T cell numbers and CD4^+^/CD8^+^ ratios while negatively correlated with immune activation of PLWH. Those two types of cells played different roles in immune activation. From a microscopic perspective, the factors affecting CD4^+^ T cell numbers may originate from CD4^+^ T cells or surrounding cells. In CD4^+^ T cells, Cai et al. observed that the down-regulation of TCF1 during HIV infection impairs T cell proliferation by destroying mitochondrial function. With the exception of CD4^+^ T cells, Qian et al. noted that the proportion of CD38^+^ CD39^+^ NK cells in HIV-infected individuals was positively correlated with HIV viral load and negatively correlated with CD4^+^ T cell numbers, with NK cells inhibiting the proliferation of CD4^+^ T and CD8^+^ T cells. In addition, based on the stemness and antiviral ability of HIV-1 specific CD8^+^ T cells expressing CXCR5, Gao et al. summarize the functions of those CD8^+^ T cells and propose strategies to translate the research findings into feasible strategies for HIV treatment and potential cure. Furthermore, in the context of an association between HIV-1 progression and non-infectious diseases, Qin et al. studied the relationship between HIV-1 and chronic kidney disease. The authors demonstrate that recovery of CD4^+^/CD8^+^ ratios was related to a lower incidence of chronic kidney disease (CKD) in HIV-1 infected patients receiving ART. Similarly, the treatment strategy based on non-nucleoside reverse transcriptase inhibitor (NNRTI) could be a better approach to restore CD4^+^/CD8^+^ ratios as well as reduce the risk of CKD.

Moving on to new strategies to eradicate HIV-1 infection, the “Shock and Kill” strategy has attracted much attention in recent years. Used in conjunction with latency reversing drugs to promote HIV-1 expression and virion production in latent cells, this strategy allows an infected individual to eliminate HIV-1 hiding within cells. Li Y. et al. synthesized a variety of compounds through the combinatorial strategy of computer-aided design and biological characterization. As a result, two agonists for TLR7/8 dual-receptors and one agonist specific for TLR8 were identified and shown to be highly efficacious in activating the latent reservoir of HIV-1 in cell lines and patient PBMCs. In particular, these agonists appeared to enhance the activity of NK and T cells.

In the late stages of a HIV infection, most HIV-infected people die from opportunistic infections due to their immune-deficient status, of which, a large proportion of deaths are caused by fungal infections. When investigating differences in lung microbiota of HIV-positive patients with different immune statuses, Chen J. et al. found that the diversity of bacteria in the lungs of HIV-positive patients was lower than that of HIV-negative patients, while the number of fungi was higher in HIV-positive patients. By studying fungal populations in the nose and mouth, Chen X. et al. discovered that invasive fungal diseases rarely developed in individuals with normal immune reconstitution, although these pathogenic filamentous fungi are readily detectable in the nose and mouth of such individuals. Meanwhile, Li Y. et al. elaborated on the neglected fungal species during HIV infections. In that context, the authors reviewed the latest progress in exploring the composition of fungal microbiota and common fungal diseases associated with HIV. The innate and adaptive antifungal immunity during HIV infections were also discussed. In addition, on the issue of intestinal microflora, Bragazzi et al.  reviewed the impact of PrEP (pre-exposure prophylaxis) on the intestinal microflora of MSM.

Early diagnosis of HIV-1 infection and early initiation of HAART are very important to achieve better virus suppression and accelerated immune reconstruction. However, new serological-based HIV-1 diagnostics and the potential for clinical application have yet to be approved. Nonetheless, Zhao et al. have developed a HIV-1 rapid recent-infection test strip (RRITS), which can distinguish HIV-1 near-term infection (RI) from long-term infection (LI) by recent infection testing algorithms (RITAs). This provides a feasible detection method for diagnosing the phase of a HIV-1 infection. In addition, identification of drug-resistant HIV-1 plays a key role in the follow-up drug treatment as well as prognosis. Li Y. et al. established a method to deep sequence the low-abundance HIV-1 drug resistance marker. This strategy is centered on NGS-based segmented amplification for detecting HIV-1 drug resistance mutations, whereby this approach can amplify the HIV-1 *pol* gene more accurately as well as economically to a threshold of 100 copies per ml.

In summary, this topic on “AIDS 40th Year” focuses on the epidemiological characteristics of HIV-1, viral differences between several HIV-1 subtypes, the consequences during pathogenesis of HIV-1 infection, the cure for HIV-1, the relationship between AIDS and other diseases, and related studies on current challenges. Collectively, findings from these studies can fill the existing gaps in certain areas of HIV/AIDS, however, more detailed studies are necessary to reduce the transmission of HIV-1, to prolong the lifespan of patients as well as to reduce patient discomfort. Finally, the development of a HIV-1 vaccine and achieving a complete cure for HIV-1 infections and AIDS still requires significant effort from researchers as well as all sectors of the community.

## Author contributions

JZ wrote the manuscript. SJ, TZ, ZC, XJ, WZ, SR-n, AK, KD, and LZ edited the manuscript.

